# ^18^ F-FDG PET standard uptake values of the normal pons in children: establishing a reference value for diffuse intrinsic pontine glioma

**DOI:** 10.1186/2191-219X-4-8

**Published:** 2014-01-28

**Authors:** Marc H A Jansen, Reina W Kloet, Dannis G van Vuurden, Sophie EM Veldhuijzen van Zanten, Birgit I Witte, Serge Goldman, W Peter Vandertop, Emile FI Comans, Otto S Hoekstra, Ronald Boellaard, Gert-Jan JL Kaspers

**Affiliations:** 1 Division of Oncology and Hematology, Department of Pediatrics, VU University Medical Center, De Boelelaan 1118, Amsterdam 1007 MB, the Netherlands; 2 Department of Radiology and Nuclear Medicine, VU University Medical Center, De Boelelaan 1117, Amsterdam 1081 HV, the Netherlands; 3 Neuro-oncology Research Group, Cancer Center Amsterdam, De Boelelaan 1117, Amsterdam 1081 HV, the Netherlands; 4 Department of Epidemiology and Biostatistics, VU University Medical Center, De Boelelaan 1118, Amsterdam 1081 HV, the Netherlands; 5 Department of Nuclear Medicine, U.L.B.-Hôpital Erasme Brussels, 808 route de Lennik, Brussels 1070, Belgium; 6 Neurosurgical Center Amsterdam, VU University Medical Center, De Boelelaan 1117, Amsterdam 1081 HV, the Netherlands

**Keywords:** Positron emission tomography, [^18^ F]fluorodeoxyglucose, Pontine glioma, Brain neoplasms, Reference values, Pons

## Abstract

**Background:**

Positron emission tomography (PET) scanning with [^18^ F]fluorodeoxyglucose (^18^ F-FDG) is a useful diagnostic and prediction tool in brain tumors, but its value in childhood diffuse intrinsic pontine glioma (DIPG) is still unclear. For interpretation of ^18^ F-FDG PET results in DIPG, uptake values of the normal pons of children of increasing ages are mandatory. The aim of this study was to determine ^18^ F-FDG standard uptake value ratios (SUVr) of the normal pons and to compare these to those of DIPG.

**Methods:**

We studied 36 subjects with a normal, non-affected pons (aged 5 to 23 years) and 6 patients with DIPG (aged 4 to 17 years) who underwent ^18^ F-FDG PET scanning. Magnetic resonance imaging (MRI) was co-registered to define the regions of interest. SUVr and SUVrmax for the pons/cerebellum (SUVr_p/c_) and the pons/occipital lobe (SUVr_p/o_) were calculated. Independent-samples *t* tests and Mann–Whitney *U* tests were used to compare the mean SUVr and Pearson’s test for correlations.

**Results:**

For the normal pons, mean SUVr_p/c_ and SUVr_p/o_ were 0.65 (±0.054) and 0.51 (±0.056), respectively. No significant correlations were found between the SUVr of the normal pons and sex, age, nor pontine volume. A modest but statistically significant correlation was found between SUVr and post-injection time acquisition timing. For DIPG, mean SUVr_p/c_ and SUVr_p/o_ were 0.74 (±0.20) and 0.65 (±0.30), respectively, while mean SUVr_p(max)/c_ and SUVr_p(max)/o_ were 1.95 (±0.48) and 1.81 (±0.20), respectively.

**Conclusion:**

The SUVr of the unaffected pons are strikingly constant between children, irrespective of sex and age, and can therefore be well used as a reference value for ^18^ F-FDG PET studies in DIPG.

## Background

Positron emission tomography (PET) scanning with [^18^ F]fluorodeoxyglucose (^18^ F-FDG) provides information on glucose metabolism. ^18^ F-FDG PET positively correlates with an increasing WHO grade in astrocytomas [1]. In high-grade glioma (HGG), ^18^ F-FDG PET is an indicator of response to therapy and is used for PET-guided planning of stereotactic brain biopsy [2-5]. In the past few years, ^18^ F-FDG PET studies have been introduced in diffuse intrinsic pontine glioma (DIPG) [6-10], a fatal disease that almost exclusively occurs in children [11]. Interestingly, ^18^ F-FDG metabolism in the majority of the DIPG was lower than that in the non-affected occipital lobe, but increased ^18^ F-FDG uptake correlated with decreased overall survival [10]. However, reference values of ^18^ F-FDG uptake in the normal pons of children of increasing age are mandatory to know what increased uptake is in the pons, and these data are lacking. Therefore, the aim of this study was to calculate the standard uptake value ratios (SUVr) for the pons/cerebellum (SUVr_p/c_) and for the pons/occipital lobe (SUVr_p/o_) in subjects with a normal pons and to investigate the influence of age, pontine size, and post-injection interval on the SUVr. The SUVr of the normal pons were then compared to the SUVr and SUVrmax of DIPG.

## Methods

### Subjects

To study the ^18^ F-FDG uptake of the normal pons, a retrospective cohort was used. Thirty-six children and adolescents aged 6 to 23 years who underwent ^18^ F-FDG PET scans for epilepsy surgery planning in the period of 2002 until 2012 were included. All controls had focal epilepsy and were in a non-ictal state at the moment of scanning. We inventoried the anti-epileptic agents used at the day of scanning. We excluded scans that revealed space-occupying lesions anywhere in the brain or epilepsy-induced changes in the pons, occipital lobe, and cerebellum and scans that did not meet the criteria as described under ‘Scanning procedure’. The affected population consisted of six children with a newly diagnosed DIPG, based on criteria as described elsewhere from VU University Medical Center (VUmc), Amsterdam, the Netherlands, who underwent an ^18^ F-FDG PET scan at diagnosis [11]. The study was approved by the institutional review board of VUmc.

### Scanning procedure

Scans of controls and DIPG patients were performed using an ECAT EXACT HR + PET scanner (Siemens/CTI, Knoxville, TN, USA), as previously described [12]. Patients and controls fasted for at least 4 h before the PET scan. Fifteen minutes before injection, they were positioned in a quiet, darkened room, with their eyes closed and no noise. After injection of 185 MBq ^18^ F-FDG (mean 187.2 MBq ±5.6), subjects remained in the quiet, darkened room for 35 min followed by a 10-min 2D transmission scan, acquired using retractable rotating ^68^Ge sources, used for attenuation correction purposes. Approximately 45 min post-injection, a static 3D emission scan of 15 min was acquired. All emission scans were reconstructed using ordered subset expectation maximization (OSEM, 4 iterations, 16 subsets) with a Hanning filter with a cutoff at 0.5 times the Nyquist frequency and included the usual corrections for normalization, decay, dead time, attenuation, scatter, and randoms [13]. During reconstruction, a zoom factor of 2.123 and a matrix of 256 × 256 were used, resulting in voxel sizes of 1.2 × 1.2 × 2.4 mm^3^. All subjects underwent structural magnetic resonance imaging (MRI) T1-T2 for diagnostic purposes. PET characteristics are summarized in Table 1.

**Table 1:** Baseline and PET characteristics of controls and patients with DIPG

	**Controls**	**DIPG**
Number of subjects	36^a^	6
Male	21	2
Female	15	4
Median age (years)	12 (±4)	6 (±5)
0 to 5	0	2
6 to 10	15	3
11 to 15	14	0
16 to 20	5	1
20 to 25	2	0
Anti-epileptic drugs		
Valproic acid	3	0
Clobazam	4	0
Carbamazepine	12	0
Levetiracetam	6	0
Lamotrigine	9	0
Other	3	0
Histology		
Anaplastic astrocytoma		2 (biopsy)
Glioblastoma multiforme		1 (autopsy)
DIPG histology unknown		3
PET characteristics		
Mean ^18^ F-FDG dose (MBq)	187 (±11)	170 (±29)
Mean scan duration (min)	15 (±0)	16 (±2)
15 min	35	5
20 min	1	1
^18^ F-FDG uptake interval time		
Mean (min)	48 (±16)	50 (±27)
PET reconstruction parameters		
Method	OSEM	OSEM
Matrix 256	34	6
Matrix 128	2	0

OSEM, ordered subset expectation maximization. ^a^The controls consisted of 6 subjects with temporal lobe, 1 with parietal lobe, and 2 with frontal lobe epileptogenic foci; 5 with hypometabolism of the hippocampus; 5 with focal cortical dysplasia; 3 with mesial temporal sclerosis; and 14 without structural or ^18^F-FDG PET epileptogenic foci.

### Image analysis

**Figure 1:**
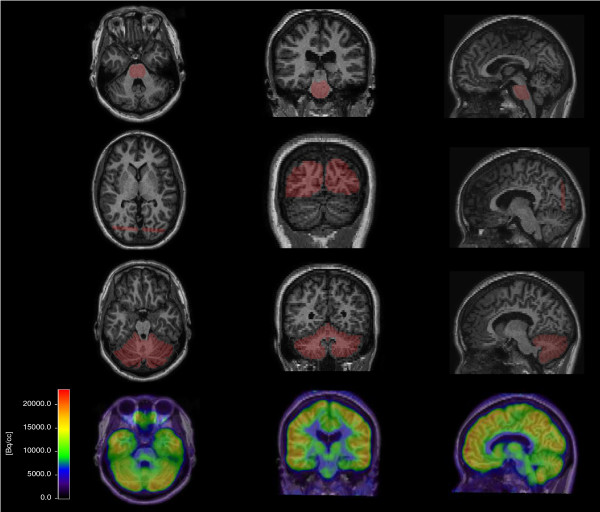
**Co-registered T1-MR and FDG PET of a control.** The ROI was defined on the co-registered T1-MR on sagittal, coronal, and axial slices. The upper row shows the ROI of the pons, the second row of the occipital lobe, and the third row of the cerebellum. For the occipital lobe, five slices were taken as the ROI from the coronal angle. The lower row shows the PET scan after T1-MRI fusion.

Each patient’s T1-weighted MR image was co-registered to their ^18^ F-FDG PET using VINCI software (Max Planck Institute, Cologne, Germany) and subsequently used to manually define the regions of interest (ROIs) of the pons, occipital lobe, and cerebellum in normal subjects (Figure 1). For DIPG, the ROI was defined as the hypointense pontine lesion on T1 MRI, independent of contrast enhancement. The ROIs were projected on the PET, and the mean uptake (becquerel per cubic centimeter) was calculated for the entire defined ROI. Next, the SUV ratios were calculated by dividing the activity (becquerel per cubic centimeter) of the pons by the reference regions. Control group reference regions were the occipital lobe (SUVr_pons/occipital_ = SUVr_p/o_) and cerebellum (SUVr_pons/cerebellum_ = SUVr_p/c_). Temporoparietal lobe was excluded as a reference region in this control group as FDG uptake may have been affected by epilepsy-induced changes in this region. For DIPG, the maximal SUV ratios (SUVr_p(max)/c_ and SUVr_p(max)/o_) were calculated by dividing the hottest pixel of the pons (becquerel per cubic centimeter) by the mean uptake of the reference region (becquerel per cubic centimeter). Finally, SUV ratios were correlated to post-injection time, age, sex, and pontine volume (calculated on MRI) in the control cohort.

### Statistics

SPSS 18.0 for Windows was used for statistical analyses. The range and distribution of the SUVr_p/c_ and SUVr_p/c_ are illustrated in histograms and boxplots. To determine whether the observations followed a normal (Gaussian) distribution, histograms and QQ plots were established. The mean, standard deviation, and corresponding confidence intervals were calculated accordingly. Based on a Gaussian distribution in both groups, independent-samples *t* tests were used to compare the mean SUV ratios of male versus female subjects. Non-parametric tests (Mann–Whitney *U* tests) were used to compare the SUVr of DIPG versus the SUVr of controls. Pearson’s correlation test was used to correlate parameters with SUV ratios.

## Results

### Baseline characteristics

Baseline characteristics are summarized in Table 1.

### SUV ratios of the normal pons

**Figure 2:**
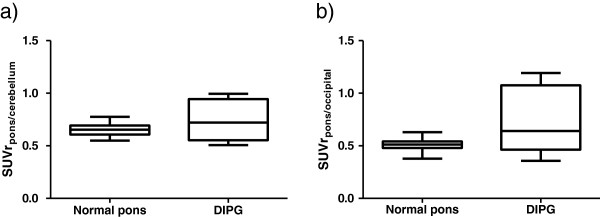
**Boxplots of SUVr**_**p/c **_**(a) and SUVr**_**p/o **_**(b) for the normal pons versus DIPG.** The SUVr deviation between controls is limited compared to that between patients with DIPG. The mean SUVr_p/c_ and SUVr_p/o_ are both not significantly higher in DIPG compared to controls. In the majority of the DIPG patients, the SUVr_p/c_ and SUVr_p/o_ are less than 1.0. Some patients with DIPG even show SUVr at the lower end of the SUVr of controls.

Controls showed consistent SUV ratios of the normal pons: a mean SUVr_p/c_ of 0.65 (±0.054) and a mean SUVr_p/o_ of 0.51 (±0.056). SUVr_p/c_ and SUVr_p/o_ showed normal Gaussian distributions as confirmed by histograms and QQ plots (Additional file 1: Figure S1). Figure 2 shows the SUV ratios of the normal pons.

### Pontine SUV ratios in relation to pontine volume, sex, and age

**Figure 3:**
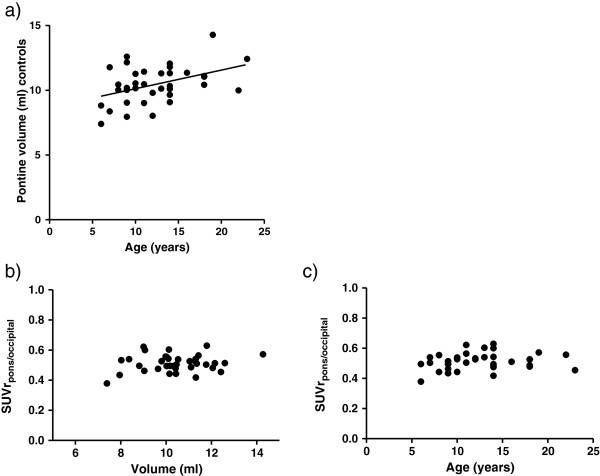
**Correlation between SUVr and pontine volume, sex, and age.** Age is significantly correlated with the pontine volume of controls as measured on MRI **(a)**. The line shown is the regression curve. The SUVr_p/o_ of controls and DIPG is plotted against pontine volume **(b)** and age **(c)**. No correlation was found between SUVr_p/o_ and these parameters. This also applies to SUVr_p/c_ (figures not shown).

The average volume of the normal pons was 10 cm^3^ (±1.4). The pontine volume linearly increased with age (regression coefficient 0.17, *r* = 0.51, *p* = 0.001; Figure 3a). There was no significant correlation between SUVr_p/o_ (*r* = 0.18, *p* = 0.28; Figure 3b) and pontine volume nor SUVr_p/c_ (*r* = −0.13, *p* = 0.45) and pontine volume. Furthermore, SUV ratios were found to be age independent, with *r* values of −0.17 (*p* = 0.324) and 0.18 (*p* = 0.305) for SUVr_p/c_ and SUVr_p/o_ (Figure 3c), respectively. We also found no significant difference between male and female subjects for SUVr_p/c_ (*p* = 0.86) nor SUVr_p/o_ (*p* = 0.98).

### Pontine FDG SUV ratios as a function of post-injection uptake time

**Figure 4:**
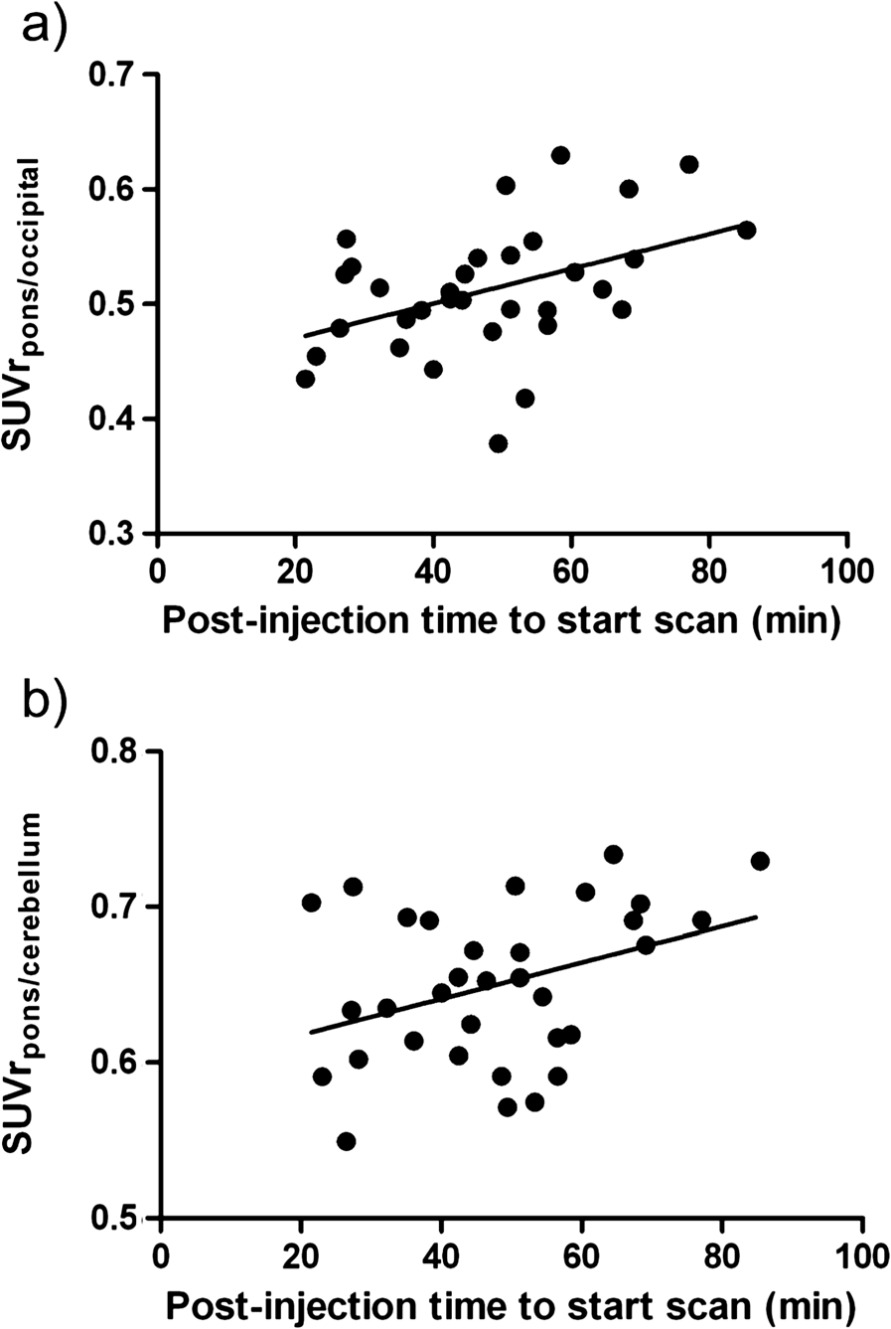
**Correlation between SUVr and post-injection (PI) time.** The SUVr_p/o_**(a)** and SUVr_p/c_**(b)** are plotted against the PI time. Both SUV ratios slightly increase over time; in other words, the pons shows a delayed uptake of ^18^ F-FDG compared to the cerebellum and occipital lobe. The line shown is the regression curve.

To determine whether uptake time influenced the ^18^ F-FDG uptake, we investigated the correlation between the SUV ratios and the post-injection uptake time in the control group (Figure 4). A modest positive correlation was found with both SUVr_p/c_ (*r* = 0.37, *p* = 0.034) and SUVr_p/o_ (*r* = 0.43, *p* = 0.012) and increasing post-injection time. The regression coefficients were small (0.0011/min and 0.0015/min, respectively).

### ^18^ F-FDG uptake in the normal pons versus DIPG

The average DIPG volume on MRI was 27 cm^3^ (±4.1). The mean SUVr_p/c_ in DIPG patients was 0.74 (±0.20), whereas in controls a SUVr_p/c_ of 0.65 (±0.054) was found (*p* = 0.64) (Figure 2). The mean SUVr_p/o_ in DIPG patients was 0.65 (±0.30), which was 0.51 (±0.056) in controls (*p* = 0.37). In only one out of six DIPGs, a SUVr_p/o_ and SUVr_p/c_ ≥1.0 was found. In three patients with increased local ^18^ F-FDG tumor uptake, the SUVrmax was calculated. The mean SUVr_p(max)/o_ was 1.81 (±0.20) and SUVr_p(max)/c_ was 1.95 (±0.48) which was significantly higher than the mean SUVr of the normal pons (*p* = 0.042 and *p* = 0.005).

## Discussion

In an era where numerous drug trials in DIPG are ongoing or will be initiated shortly, it is essential to develop tools to predict disease evolution and to monitor response to therapy [14]. ^18^ F-FDG PET has the potential to be such a tool. However, the interpretation of ^18^ F-FDG PET results in DIPG is hampered by a lack of data on normal pontine glucose metabolism in children. We show in this study that ^18^ F-FDG SUV ratios of the normal pons versus those of the cerebellum and occipital lobe are very consistent in between controls, independent of sex, age, and pontine volume, and are therefore suitable as a reference value for ^18^ F-FDG PET studies in DIPG. Not only the pons of controls but also the pons infiltrated by tumor often showed lower ^18^ F-FDG uptake than the cerebellum and occipital lobe, a phenomenon that has been reported before [10]. Moreover, the mean SUVr of DIPG were not significantly higher than those of the normal pons, but this is probably due to the small DIPG sample size as the standard deviations were high. One may therefore question the role of ^18^ F-FDG PET in DIPG; however, the mean SUVrmax clearly increased in DIPG compared to the normal pons. Indeed, a recent study showed a significant correlation between increased ^18^ F-FDG tumor uptake and decreased survival in patients with this disease [10]. This correlation might be even stronger when considering that a SUVr_p/o_ in DIPG between 0.5 and 1.0 already reflects increased ^18^ F-FDG uptake in comparison with the normal pons. This consideration is not taken into account in studies using semi-quantitative measurements that lead to classification as ‘hypo/iso/hypermetabolic’ compared to other brain areas [6-10].

An explanation for the limited ^18^ F-FDG uptake in DIPG compared to supratentorial HGG is that DIPGs are heterogeneous tumors with a mixed histologic tumor grade, as local uptake of the tracer is related to the presence of anaplastic features [11,15,16]. Calculating the SUVrmax, reflecting the highest local uptake in the tumor, is helpful in those tumors with heterogeneous ^18^ F-FDG uptake. Other explanations of the limited uptake are the frequently observed integrity of the blood–brain barrier in DIPG and the presence of white matter in the pontine region, which has low glucose metabolism [17].

We further investigated whether the time between injection and PET scanning had an influence on the ^18^ F-FDG uptake in the pons of controls compared to other brain areas. Indeed, SUVr_p/c_ and SUVr_p/o_ were positively correlated with increasing post-injection time. This suggests a delayed uptake of this tracer in the pons compared to the cerebellum and occipital lobe. However, the SUVr regression coefficients were small, and therefore, the influence of the uptake interval in clinical practice is negligible.

The main advantage of SUV ratios is that the possible errors in the measurement of weight or transcription and dose administered are minimized by the ratio between the two SUV measurements [18]. This applies especially for pediatric cancer, with low patient numbers and therefore often multi-national multi-center trials. In this study, we showed that SUV ratios of the normal pons are independent of sex, pontine volume, and age, although we had an under-representation of the youngest children (<5 years) in the control group. Although SUV ratios may give useful information in serial measurements, they have their limitations. In situations in which the ^18^ F-FDG uptake of the reference tissue varies, changes in SUV ratios can be misleading. For example, this may be the case when patients use steroids, which influence the glucose metabolism of the brain [19]. A methodological issue in this study was the use of epilepsy patients as controls, as ^18^ F-FDG PET data of healthy children could not be obtained due to ethical reasons regarding radiation exposure. We, however, do not expect significant changes in glucose metabolism of the pons due to epilepsy as all our subjects were in an inter-ictal state, which is not associated with changed glucose metabolism [20]. Furthermore, several anti-epileptic drugs including phenobarbital, phenytoin, benzodiazepines, and valproic acid have been associated with hypometabolism of the brain and especially the cerebellum and may therefore overestimate the SUVr_p/c_. Of these drugs, only valproic acid and clobazam were used in this study by, respectively, 3 and 4 out of 37 controls [21,22]. The lack of variance in between controls of both SUVr_p/c_ and SUVr_p/o_ presumes that the use of anti-epileptic drugs has not influenced our results significantly. In addition, the use of the cerebellum as a reference in epileptic patients in ^18^ F-FDG PET studies is not uncommon [23,24].

Future ^18^ F-FDG PET studies in DIPG may now compare SUVr and SUVrmax in DIPG to the here reported mean SUV ratios of the normal pons. By comparing SUV ratios to the normal pons, smaller increases in glucose metabolism can be detected in comparison with semi-quantitative measurements, as DIPGs often show lower glucose metabolism than the reference brain tissue (occipital lobe). In this way, the sensitivity and applicability of ^18^ F-FDG PET as a predictive and response monitoring tool for patients with DIPG can be increased.

## Conclusion

We established a reference SUVr for ^18^ F-FDG uptake in the normal pons. SUV ratios are very consistent in between controls and independent of pontine volume, sex, or age. Not only was the ^18^ F-FDG uptake in the normal pons low compared to that in the reference brain areas, but also the uptake in DIPG was often lower than that in the occipital and cerebellar tissues. We encourage a study in controls to validate our results and propose that future ^18^ F-FDG PET trials in DIPG calculate SUV and SUV(max) ratios in order to relate these to the here reported mean SUV ratios of the normal pons. Smaller changes in the tumor’s glucose metabolism can be detected in this way, which may have prognostic relevance for the patient.

## Competing interests

The authors declare that they have no competing interests.

## Authors’ contributions

MJ, RK, OH, RB, DV, GK, and WV contributed to the concept and study design. MJ, RK, OH, EC, SG, and SV collected the data. MJ and BW performed the statistical analysis. MJ, RK, OH, RB, GK, and BW were involved in the interpretation of the data. All authors were involved in the writing process and all approved the manuscript before submission.

## Supplementary Material

Additional file 1: Figure S1 SUVr_p/c_ and SUVr_p/o_ distributions in normal controls. Normal Gaussian distributions of both SUVrs are presented in histograms (a, d) and boxplots (b, e). The Gaussian distribution was confirmed by QQ plots (c, f). Click here for file
